# Selection biases in elite youth handball: early maturation compensates for younger relative age

**DOI:** 10.3389/fspor.2025.1579857

**Published:** 2025-04-28

**Authors:** Lutz Thieschäfer, Jörg Schorer, Jochen Beppler, Dirk Büsch

**Affiliations:** ^1^ Sport and Training Department, Institute of Sport Science, School of Humanities and Social Sciences, Carl von Ossietzky Universität Oldenburg, Oldenburg, Germany; ^2^ Sport and Movement Science Department, Institute of Sport Science, School of Humanities and Social Sciences, Carl von Ossietzky Universität Oldenburg, Oldenburg, Germany; ^3^ Deutscher Handballbund e.V., Dortmund, Germany

**Keywords:** adolescent, biological age, development, maturity, relative age effect, secondary data, talent

## Abstract

Talent selections in youth sports are frequently biased regarding the maturation and relative age of the players, with preference given to more mature and relatively older players. It thus can be hypothesized that relatively younger players born at the end of the selection year must mature earlier to compensate for this disadvantage. Hence, this study investigated maturation, relative age, and their association in the talent selection of German youth handball players. A secondary data analysis within an ex post facto design was conducted to examine the birth quarter distributions and maturation parameters of 2,259 female U15 players and 2,340 male U16 players. Practically significant maturation bias was detected in male players, who matured almost one year earlier than common German boys (*g* = −1.67). This was not evident in female players. Relative age selection biases were observed in female (פ‎ = .16) and male (פ‎ = .20) players. An analysis of maturation timing across birth quarters revealed that relatively younger players born later in the selection year mature earlier than their relatively older peers in both female (*g* = 0.99) and male players (*g* = 0.56), thereby partially offsetting relative age disadvantages. Consequently, it may be crucial for relatively younger players to be early-maturing to increase selection odds. Considering the evidence indicating the presence of both maturation and relative age selection biases, it seems prudent to acknowledge the significant impact that these can have on talent selection and development in German youth handball. The development of solutions is currently underway in collaboration with the regional and national handball federations.

## 1 Introduction

National sports federations employ structured talent identification and development (TID) systems in youth sports with the objectives of identifying young athletes with the greatest potential for long-term success and preparing them for the challenges they will encounter in adult international competition (1, 2). The players selected for the development system benefit from the provision of professional coaching, sports science, and medical support, gain access to superior training equipment and facilities, and are exposed to high levels of competitive challenge to facilitate their long-term progress and increase the likelihood of attaining success at the senior level (3). The German Handball Federation (DHB; Ger.: Deutscher Handballbund) annually conducts scouting camps at the beginning of the year to select female U15 and male U16 players for talent development programs and to recruit them for the youth national squads (4). Each coach of the 20 regional handball federations in Germany selects out of their regional pool approximately twelve female and twelve male players across all playing positions who are perceived as “talented” and sends them as one female and one male team to the National Talent Selection Camp of the German Handball Federation (SelCamp; Ger.: DHB-Sichtung). Due to organizational considerations, the five-day SelCamp is conducted on two consecutive occasions, with ten teams participating in each session for each sex (four three-day SelCamps, with five teams each for each sex since 2024). During this selection process, candidates are required to demonstrate their abilities in general motor tests, their skills in handball-specific tests as well as their technical and tactical qualities in matches and competitions. Based on these observations, the national coaches then select the most promising players to participate in the National Talent Nomination Camp of the German Handball Federation (NomCamp; Ger.: 1. DHB-Lehrgang), which ultimately serves as the recruitment pool for the youth national team (4). However, two nonmodifiable factors that can bias these (pre)selections are the biological maturation of the players as well as their relative age (5–10).

The process of biological maturation refers to the progression toward a fully developed mature stature. It can be characterized in three ways: the maturation stage at the time of observation (i.e., maturity status), the age at which specific maturational events occur (i.e., maturity timing), such as the age of peak height velocity (APHV), and the rate at which maturation occurs (i.e., maturity tempo) (7, 11). An advanced biological maturity status may result in advantageous anthropometric (e.g., body height, wing span) and physical (e.g., strength, power, endurance) characteristics, which are considered to underpin high sports performance (12, 13). It is thus unsurprising that an advanced maturity status has been shown to increase selection odds in sports where greater size, strength, and power are desired attributes, which in turn promotes biological maturation selection bias (6–10, 14–20). This phenomenon is not unprecedented in sports; rather, it has been documented for several decades (21). However, while early/advanced maturation may confer an initial advantage in highly physical sports, it does not necessarily translate to success at the senior level. In fact, it has been suggested that late-maturing players may perform better in adulthood when retained in TID systems (22, 23).

Recent observations in handball players indicate that an advanced biological maturity status results in superior anthropometrical and physical performance characteristics (13). Moreover, early-maturing handball players have been shown to exhibit advantages in anthropometry, strength, speed, and jump performance compared with their later-maturing peers (19, 24). Consequently, handball may be susceptible to a maturation selection bias. This is supported by data obtained by de la Rubia et al. (19), Tróznai et al. (14), and Tróznai et al. (20). De la Rubia et al. (19) reported an overrepresentation of early-maturing players in U16 and U17 Spanish academy handball. Tróznai et al. (14) observed a mean difference (with standard deviations in parentheses) between bone age and chronological age (as a proxy for maturity status) of 0.9 (1.1) years in female U14 and 1.8 (1.0) years in male U15 Hungarian handball players which indicates a bias toward players exhibiting an advanced maturity status. The data provided by Tróznai et al. (20) further indicates that the difference between bone age and chronological age increases with rising selection level.

In addition to maturation, player selections can also be biased regarding the relative age of the players. Relative age refers to chronological age differences between individuals within the same age cohort, and its consequences are known as relative age effects (25, 26). For example, an individual born close to the cutoff date at the beginning of the selection year (e.g., 1st January) is almost 12 months older than their peers born at the end of the selection year (e.g., 31st December) and therefore relatively older. Although annual age grouping in youth sports is employed to maintain general developmental similarities among players within the same age group to allow for more balanced coaching and evaluation as well as equal and fair competition, it can still provide disadvantages to some of the group members, causing relative age effects (12, 27). These effects typically manifest themselves in selections as an underrepresentation of players born at the end of the selection year (i.e., relatively younger players) and an overrepresentation of players born at the beginning of the selection year (i.e., relatively older players) (12). Several mechanisms have been proposed to account for this phenomenon, with differences in maturation primarily held responsible for the development of relative age effects (12, 26). It has typically been suggested that relatively older children have an increased probability of being physically more mature and entering puberty earlier than their relatively younger peers due to their advanced chronological age (26). During circumpubertal ages in particular, an age difference of almost a year can result in significant differences in physical qualities (26, 28). The maturation-selection hypothesis, which is frequently invoked when elucidating relative age effects, posits that coaches may confuse these maturation-dependent anthropometrical and physical advantages with “talent”, increasing the likelihood of selecting relatively older players, which represents a common relative age selection bias (27, 29). Nevertheless, it is important to note that relatively older players are not inherently more advanced in their maturation in comparison to their relatively younger peers because maturation individually varies in terms of timing and tempo (11). Therefore, relative age and biological maturation are regarded as distinct constructs and should be treated as such (5, 9, 30). A substantial body of research has documented the existence of relative age selection biases in handball at the youth level. These studies involved players of both sexes on national teams, club teams, and tournaments across various countries, including Brazil (31, 32), Denmark (33), Germany (27, 34–37), Hungary (14, 20), Israel (38), Kosovo (39), Norway (40), and Spain (41–46). While an older relative age may provide certain advantages at the junior level, studies suggest that under certain conditions, athletes of younger relative age who successfully overcome the relative age selection bias could be superior to their peers on various performance indicators at the senior level (37, 47).

Given that both relatively older and early-maturing players are often favored in selection processes at the youth level, it could be assumed that relatively younger selected players born at the end of the selection year tendentially mature earlier and have a similar maturity status as their relatively older peers to compensate for their younger relative age. This phenomenon has been documented in soccer (9, 16, 18, 48–55), handball (14, 20, 24), tennis (56), swimming (57), and winter sports (54, 58, 59), although some conflicting results have also been reported (60). Three studies could be identified that investigated the associations between relative age and maturation in handball: Matthys et al. (24) reported no statistically significant differences in maturation timing between relatively older and younger 14-year-old male Belgium handball players. The studies conducted by Tróznai et al. (14) and Tróznai et al. (20) demonstrated descriptively, albeit not statistically significant, that relatively younger male players may exhibit greater differences between bone age and chronological age than relatively older players. This finding suggests that male selected players who are relatively younger may have an advanced maturity status. However, this was not evident in female players. In view of the inconclusive results obtained in handball, which did not statistically show the same effects as observed in other sports, it was deemed worthwhile to test a larger sample to achieve greater test power.

Accordingly, this study aimed to investigate three key areas: first, to examine potential maturation selection biases; second, to investigate potential relative age selection biases; and third, to examine the associations between maturation and relative age. Based on the findings presented above, we separately hypothesized that maturation and relative age selection biases would be present in the selection of handball players who were chosen to participate in the SelCamp (i.e., at the regional selection level), with earlier maturing players and relatively older players being in favor. It was further hypothesized that relatively younger selected players born in later birth quarters mature earlier to overcome potential relative age disadvantages. Given the potential influence of selection level on maturation and relative age selection biases (20), the subsequent selection for participation in the NomCamp (i.e., at the national selection level) was additionally considered, albeit in an exploratory manner.

## 2 Methods

The study employs an ex post facto design with a secondary data analysis, which is exempt from ethics approval because of the previously conducted collection and retrospective analysis of anonymized data. Good practice standards for conducting secondary data analyses were followed (61).

### 2.1 Subjects

The DHB provided a dataset comprising birthdate, body mass, body height, and sitting height of *n* = 2,259 female U15 players and *n* = 2,340 male U16 players who participated in the annual SelCamp from 2010 to 2020 [the mean chronological ages were 14.71 (0.28) years and 15.74 (0.28) years, respectively]. Of those players, *n* = 498 female players and *n* = 489 male players were eventually selected for participation in the NomCamp.

Players who were either younger or older than those in the regular cohort, which is U15 for girls and U16 for boys, were not included in the dataset. It is important to note that the players who participate in the SelCamp have been selected by the coaches of their respective regional handball federation (i.e., at the regional selection level). However, the player selections for participation in the NomCamp were determined by the national coaches (i.e., at the national selection level) (4).

### 2.2 Procedures

In German handball, annual age grouping is determined by the dates from 1st January to 31st December (34). Consequently, birth months were extracted from the birthdates and categorized as follows: January–March = birth quarter 1 (Q1), April–June = birth quarter 2 (Q2), July–September = birth quarter 3 (Q3), and October–December = birth quarter 4 (Q4). The chronological age was determined as the age at the date of the SelCamp.

Data of players’ anthropometry (i.e., body mass, body height, and sitting height) and chronological age were utilized to noninvasively estimate maturity offset [time difference from peak height velocity (PHV)] based on the sex-specific (modified) (62) equations proposed by Mirwald et al. (63). The estimated APHV was calculated as the difference between the maturity offset and the chronological age. The maturity offset is considered the current maturity status, and the APHV represents the timing of the growth spurt, with a lower APHV indicating earlier maturation. Players’ birthdates were known to the regional and national coaches, but only the national coaches had information on their maturity status.

### 2.3 Statistical analysis

Statistical analyses were performed with R 4.4.1, IBM SPSS Statistics (29.0.0.0, IBM, Armonk, NY, USA), and G*Power 3.1.9.7 (64). Owing to the large sample size, the statistical significance level was set to *α* = .001. APHV and maturity offset were determined to be the operationalized dependent variables.

The presence of maturation selection biases was evaluated by conducting one-sample *t*-tests to compare the mean APHV of female and male players with the mean APHV of German girls of 12.00 (0.88) years and German boys of 14.07 (0.98) years (65). Hedges’ *g* effect sizes with 95% CIs were calculated and interpreted as described below.

To examine relative age selection biases, chi-square goodness-of-fit tests were performed to determine whether the distributions of the birth quarters differed from the expected uniform distribution (26). The effect size Fei (פ‎) (66) and 90% CIs were calculated to determine the magnitude of difference in frequency counts and interpreted on the scale of a correlation coefficient according to the guidelines of Funder and Ozer (67) with thresholds of <.05, .05–.09, .10–.19, .20–.29, .30–.39, and ≥.40 as tiny, very small, small, medium, large, and very large effects, respectively. Odds ratios with 90% CIs for Q1 vs. Q4 were additionally calculated as a tangible effect size measure.

One-way analyses of variance (ANOVA) were performed to examine whether maturity offset and APHV differed between the birth quarters. Shapiro‒Wilk and Levene tests were performed in advance to test for normal distribution and homogeneity of variance. Planned contrasts analyses were conducted for the APHV, with weights of 1, 1, −1, and −1 across the birth quarters to test the hypothesis that players born in later birth quarters mature at an earlier age than players born in earlier birth quarters. Effect sizes with confidence intervals were calculated. An orientation of small, medium, and large effects was based on *η*^2^ values of .01–.05, .06–.13, and ≥.14 and Hedges’ *g* values of 0.20–0.49, 0.50–0.79, and ≥0.80, respectively (68).

In an exploratory analysis, all statistical procedures were repeated with the players selected for participation in the NomCamp to ascertain whether any potential biases persist at the subsequent national selection level.

## 3 Results

### 3.1 Maturation selection biases

**Figure 1:**
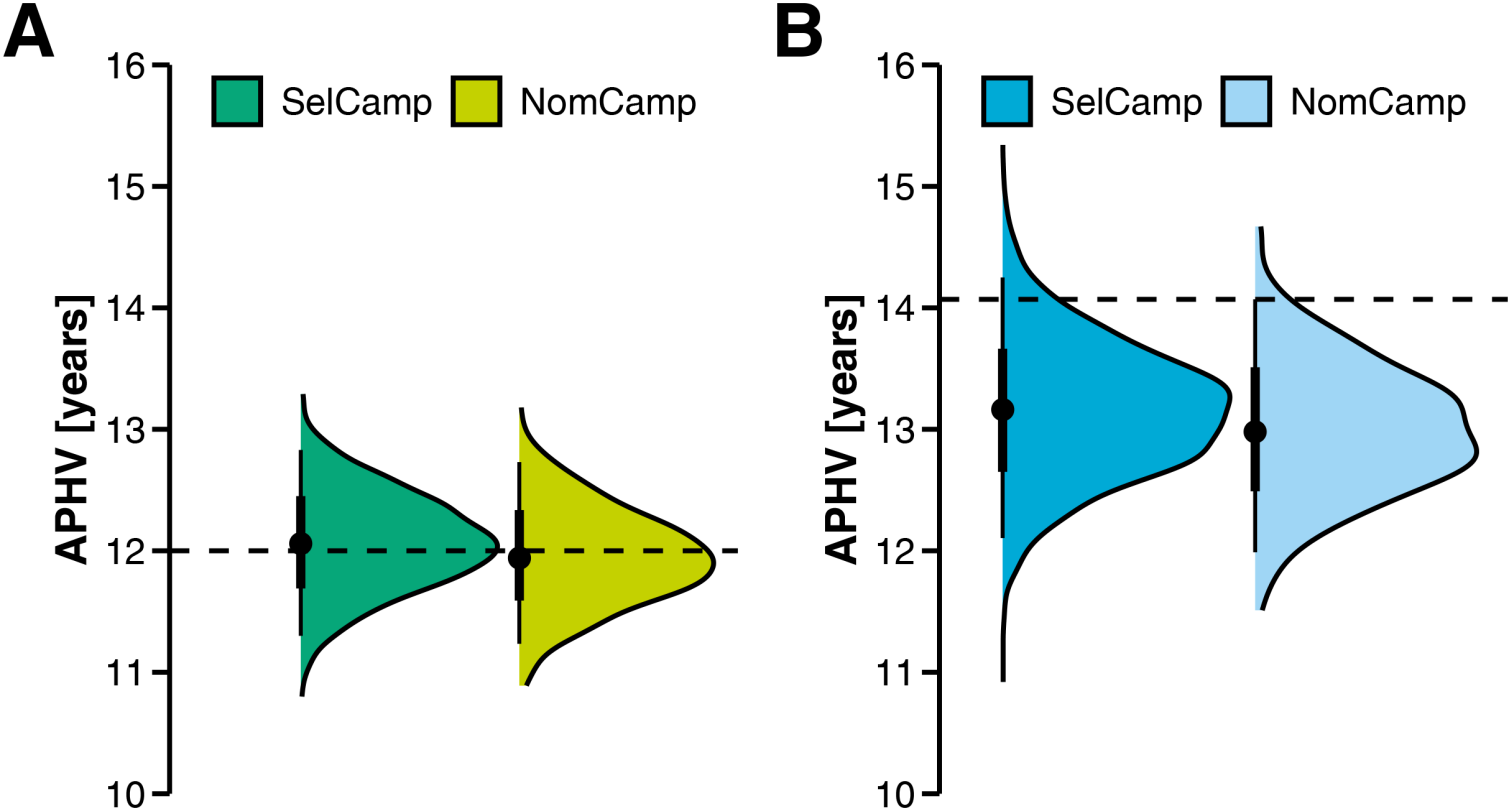
Age at peak height velocity (APHV) in female and male players. Panel **(A)**: Selections of female players. Panel **(B)**: Selections of male players. The dashed lines indicate the mean APHVs of German girls and boys (65). NomCamp = National Talent Nomination Camp of the German Handball Federation; SelCamp = National Talent Selection Camp of the German Handball Federation.

The APHVs of female and male players at the SelCamp and NomCamp are illustrated as half-eye plots in Figure 1. The mean APHV of 12.07 (0.39) years observed in female players at the SelCamp statistically significantly differed from the mean APHV of 12.00 (0.88) years observed in common German girls (65), but with an effect size below the “small” category, *t*(2,258) = 8.36, *p* < .001, *g* = 0.18, 95% CI [0.13, 0.22]. Conversely, at the NomCamp, the mean APHV of 11.95 (0.39) years was not statistically different from the reference sample (65), *t*(497) = −3.02, *p* = .003, *g* = −0.14, 1-*β* = .43.

Whereas, the mean APHV of 13.16 (0.55) years in male players at the SelCamp was significantly lower than the mean APHV of 14.07 (0.98) years observed in a representative sample of German boys (65) with a large effect size, *t*(2,339) = −80.77, *p* < .001, *g* = −1.67, 95% CI [−1.73, −1.61]. Comparable results were found at the NomCamp, with a lower mean APHV of 13.00 (0.54) years, *t*(488) = −44.16, *p* < .001, *g* = −1.99, 95% CI [−2.15, −1.84].

### 3.2 Relative age selection biases

**Figure 2:**
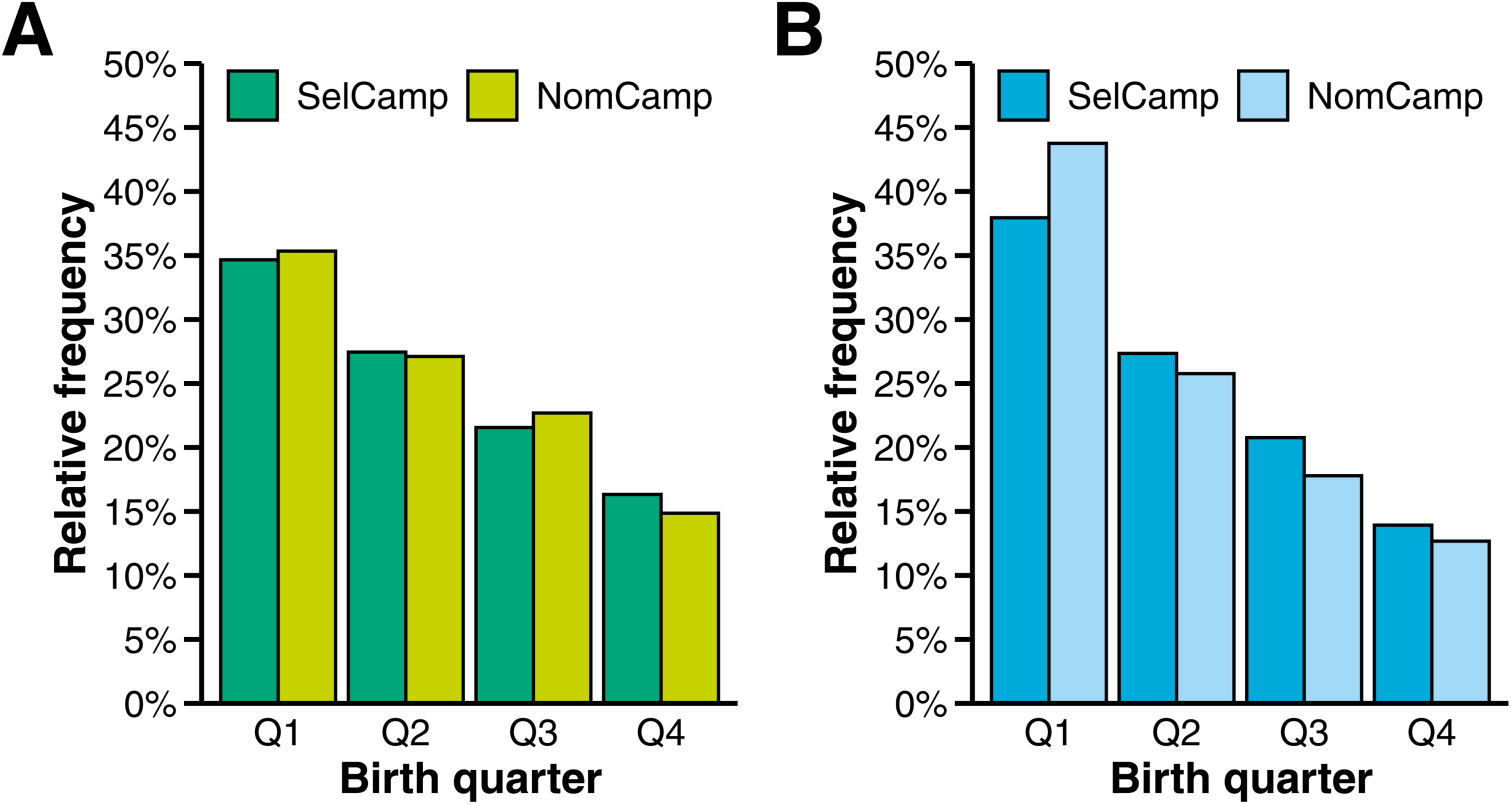
Birth quarter distributions in female and male players. Panel **(A)**: Selections of female players. Panel **(B)**: Selections of male players. NomCamp = National Talent Nomination Camp of the German Handball Federation; SelCamp = National Talent Selection Camp of the German Handball Federation.

Figure 2 illustrates the distributions of birth quarters for female and male players. Irrespective of sex and selection level, the majority of players were born during the first quarter, with the number of births declining in subsequent quarters. The distribution of birth quarters showed a significant difference from a uniform distribution in female players at the SelCamp, indicating a relative age selection bias with a small effect size, *χ*^2^(3) = 168.30, *p* < .001, פ‎ = .16, 90% CI [.14, .18]. The odds ratio calculations revealed that the odds of being born in Q1 were more than twice as high as those of being born in Q4, *OR* = 2.72, 90% CI [2.41, 3.06]. Similar results were obtained at the NomCamp, *χ*^2^(3) = 43.74, *p* < .001, פ‎ = .17, 90% CI [.12, .21], *OR* = 3.13, 90% CI [2.42, 4.05].

A relative age selection bias with a medium effect size was observed in male players at the SelCamp, as the birth quarter distribution significantly differed from the expected uniform distribution, *χ*^2^(3) = 293.53, *p* < .001, פ‎ = .20, 90% CI [.18, .22]. The odds of being born in Q1 were more than three times as high as those of being born in Q4, *OR* = 3.78, 90% CI [3.35, 4.26]. The transition to the subsequent NomCamp slightly augmented the already existing relative age selection bias, *χ*^2^(3) = 108.83, *p* < .001, פ‎ = .27, 90% CI [.23, .31], *OR* = 5.36, 90% CI [4.09, 7.01].

### 3.3 Association between maturation and relative age

**Figure 3:**
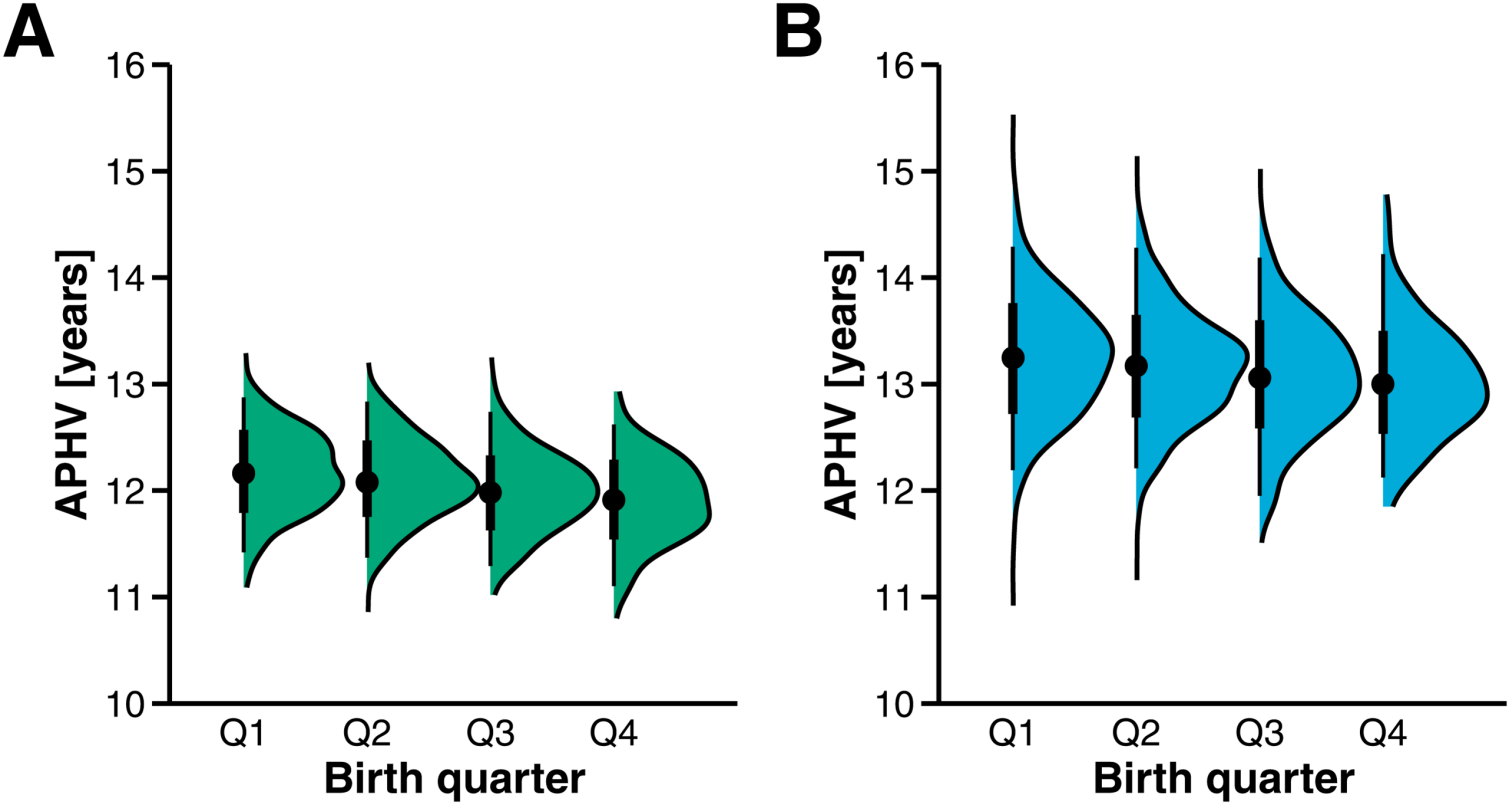
Age at peak height velocity (APHV) across birth quarters in female and male players. Panel **(A)**: Female players. Panel **(B)**: Male players.

The descriptive statistics and ANOVA results for the dependent variables are presented in Table 1 for female players and in Table 2 for male players. The APHV and maturity offset were significantly different between birth quarters in female players at the SelCamp, with medium to large effect sizes, *F*(3, 2,255) = 49.98, *p* < .001, *η*^2^ = .06, 90% CI [.05, .08], *F*(3, 2,225) = 149.37, *p* < .001, *η*^2^ = .17, 90% CI [.14, .19], respectively. Similar results were observed at the NomCamp (please see Table 1). Planned contrast analysis revealed that younger relative age is accompanied by a lower APHV, *t*(2,255) = 11.26, *p* < .001, *g* = 0.99, 95% CI [0.81, 1.16], indicating that relatively younger players mature at an earlier age, as illustrated in Figure 3.

**Table 1:** Descriptives and results of ANOVA for female players.

Camp	Variable	Q1		Q2		Q3		Q4		Total		*F*	*p*	*η* ^2^	90% CI
		*M*	*SD*	*M*	*SD*	*M*	*SD*	*M*	*SD*	*M*	*SD*				
SelCamp	Count	*n* = 783 (34.66%)		*n* = 620 (27.45%)		*n* = 487 (21.56%)		*n* = 369 (16.33%)		*n* = 2,259					
	APHV [years]	12.17	0.38	12.10	0.38	11.99	0.37	11.91	0.39	12.07	0.39	49.98	<.001	.06	[.05, .08]
	Maturity offset [years]	2.84	0.38	2.66	0.38	2.53	0.37	2.36	0.40	2.64	0.42	149.37	<.001	.17	[.14, .19]
NomCamp	Count	*n* = 176 (35.34%)		*n* = 135 (27.11%)		*n* = 113 (22.69%)		*n* = 74 (14.86%)		*n* = 498					
	APHV [years]	12.04	0.39	11.96	0.36	11.87	0.36	11.81	0.42	11.95	0.39	8.27	<.001	.05	[.02, .08]
	Maturity offset [years]	2.97	0.39	2.81	0.37	2.65	0.36	2.45	0.43	2.78	0.42	37.30	<.001	.19	[.13, .23]

APHV = age at peak height velocity; NomCamp = National Talent Nomination Camp of the German Handball Federation; Q = birth quarter; SelCamp = National Talent Selection Camp of the German Handball Federation.

**Table 2:** Descriptives and results of ANOVA for male players.

Camp	Variable	Q1		Q2		Q3		Q4		Total		*F*	*p*	η^2^	90% CI
		*M*	*SD*	*M*	*SD*	*M*	*SD*	*M*	*SD*	*M*	*SD*				
SelCamp	Count	*n* = 888 (37.95%)		*n* = 640 (27.35%)		*n* = 486 (20.77%)		*n* = 326 (13.93%)		*n* = 2,340					
	APHV [years]	13.24	0.55	13.17	0.53	13.07	0.55	13.03	0.54	13.16	0.55	17.19	<.001	.02	[.01,.03]
	Maturity offset [years]	2.78	0.55	2.60	0.53	2.44	0.56	2.24	0.53	2.58	0.58	92.24	<.001	.11	[.09,.13]
NomCamp	Count	*n* = 214 (43.76%)		*n* = 126 (25.77%)		*n* = 87 (17.79%)		*n* = 62 (12.68%)		*n* = 489					
	APHV [years]	13.10	0.55	12.98	0.50	12.85	0.54	12.89	0.51	13.00	0.54	5.61	<.001	.03	[.01,.06]
	Maturity offset [years]	2.93	0.56	2.79	0.51	2.66	0.54	2.39	0.49	2.78	0.56	17.93	<.001	.10	[.06,.14]

APHV = age at peak height velocity; NomCamp = National Talent Nomination Camp of the German Handball Federation; Q = birth quarter; SelCamp = National Talent Selection Camp of the German Handball Federation.

Significant differences in the APHV and maturity offset between birth quarters were detected in male players at the SelCamp, with small to medium effect sizes, *F*(3, 2,336) = 17.19, *p* < .001, *η*^2^ = .02, 90% CI [.01, .03], *F*(3, 2,336) = 92.24, *p* < .001, *η*^2^ = .11, 90% CI [.09, .13], respectively. Comparable outcomes were documented at the NomCamp (please see Table 2). The result of the planned contrasts analysis indicated that younger relative age was accompanied by reduced APHV, *t*(2,336) = 6.36, *p* < .001, *g* = 0.56, 95% CI [0.39, 0.74].

## 4 Discussion

This study was conducted to investigate three specific aims. The first and second aims were to examine maturation and relative age selection biases in the selections of players for participation in the SelCamp. The third objective was to determine whether maturation was associated with relative age in the selections. The hypotheses were that the selections are biased regarding maturation and relative age. It was further hypothesized that relatively younger players would mature earlier, thereby overcoming potential relative age disadvantages.

The main findings indicated the presence of a substantial maturation selection bias in male (*g* = −1.67), but not in female players (*g* = 0.18). Furthermore, in the selection process, preference was given to relatively older players (i.e., born in earlier birth quarters), with a small effect size observed in female (פ‎ = .16) and a medium effect size observed in male (פ‎ = .20) players, indicative of relative age selection biases. As hypothesized, the players born in later birth quarters matured earlier to compensate for their younger relative age, with a large effect size observed in female players (*g* = 0.99) and a medium effect size in male players (*g* = 0.56).

Although the APHV of female players at the SelCamp was statistically significantly higher than in the reference sample, the magnitude of the difference was not of practical importance. Hence, the absence of a practically meaningful maturation selection bias in female players did not confirm our hypothesis and is contrary to the abovementioned results obtained by Tróznai et al. (14). The players at the NomCamp demonstrated only a slight tendency toward earlier maturation. The absence of meaningful maturation biases toward earlier maturation could be explained by the fact that, at the time of the selections, the players were already far beyond the average PHV. Consequently, later-maturing girls have potentially made up the leeway in their physical development, thereby diminishing the initial advantages of earlier maturation. In contrast to female players, considerable maturation selection bias was evident in male players, who matured almost one year earlier than the average German boy (65), confirming our hypothesis for males. This is consistent with the data obtained by de la Rubia et al. (19) and Tróznai et al. (14). The selection of the national coaches marginally increased the already substantial bias. The preference for more mature players in male selections clearly disadvantages later maturing players. Observations from a practical perspective in German handball indicate that early-maturing players are frequently placed in the subsequent higher age group within their club. It may be assumed that the training in these groups is typically at a higher performance level, characterized by enhanced quality, and led by coaches with higher expertise. Additionally, players may also encounter higher-performing teammates and more challenging opponents at tournaments. This exposure to high-level training and competition could provide early-maturing players with an edge (10), which may, in turn, promote maturation selection bias.

The presence of relative age selection biases in this study corroborates our hypotheses and lends further support to the plethora of studies that have reported such biases in youth handball (14, 20, 27, 31–46). In female players, the probability of being born within the first quarter (Q1) than that of being born within the final quarter (Q4) of the year aligns with the *OR* of 2.29 observed by Lidor et al. (38). The relative age selection biases were not exacerbated from the SelCamp to the NomCamp, which is consistent with previous findings (14, 20, 36). In male players, the odds of being born in Q1 than that of being born in Q4 are similar to the *OR* of 2.8 documented by Doncaster et al. (42). The already existing relative age selection bias was only slightly intensified at the subsequent NomCamp. Although sex is a known moderator of relative age effects (26), the relative age selection biases were only slightly stronger in male players, which is in line with previous research (34, 36, 37). Taken as a whole, players born earlier in the selection year benefit from their higher relative age in the selection process during childhood. However, the relative age effects in handball appear to decrease in the subsequent years into adulthood and further diminish across career stages in later adult years (34, 37).

A combined analysis of maturation and relative age revealed that relatively younger female and male players tend to mature at an earlier age than their relatively older counterparts do (please see Figure 3), confirming the initial hypothesis. This phenomenon has been documented in numerous studies, albeit in sports other than handball. Previous studies conducted in handball (14, 20, 24) did not support these and present findings. In the current study, the mean APHV decreased from Q1 to Q4 by 0.26 years in female players and by 0.21 years in male players, representing the amount of compensation for relative age. Nevertheless, early maturation did not fully offset the relative age differences, and the absolute compensation was relatively modest. When the relative age difference between Q1 and Q4, which is 0.74 years in both sexes, is considered, early maturation compensates for approximately only one-third of this difference.

Given the existence of maturation and relative age selection biases at the SelCamp, the talent pool from which national coaches select their players is inherently biased. However, the rising selection level from the SelCamp to the NomCamp only marginally exacerbated maturation and relative age selection biases. These findings align with the results of recent studies in Hungarian handball that indicated that relative age selection biases did not significantly increase from the regional to the national level (14, 20).

In an effort to address the issues of maturation and relative age selection bias in elite German youth handball, measures have already been taken by the DHB, such as an early assessment of players’ biological age (biannually, starting approximately two years prior to the SelCamp) and the sensitization of coaches regarding relative age effects. Further solutions are currently being developed in cooperation with the regional and national handball federations.

### 4.1 Limitations

Readers should be mindful of the limitations of this study. Although the method proposed by Mirwald et al. (63) to noninvasively estimate maturity status in the present study is an established one, it should be noted that, as with any estimation, there may be discrepancies between the estimated and actual maturity status. According to previous validation studies by Malina et al. (69) and Kozieł and Malina (70), the equations tend to overestimate the APHV when assessing early-maturing individuals. Furthermore, the estimated APHV increases with chronological age at prediction. Thus, the players may actually mature earlier than estimated, which would increase maturation selection biases.

### 4.2 Conclusions and practical implications

This investigation identified the existence of maturation and relative age selection biases in the selections of players for participation in the SelCamp. Relatively younger players (born in later quarters of the selection year) tended to mature earlier than their relatively older peers born in preceding quarters. This suggests that younger relative age was (partially) offset by early maturation. Consequently, it may be crucial for players born in later quarters to be early-maturing to increase their likelihood of overcoming relative age selection bias and being perceived as a “talent”. Players who mature at a later age and are born in later quarters face a dual disadvantage when competing with more mature and relatively older players.

Eventually, regardless of whether players are not selected due to their later maturation or their younger relative age, players are unfavored probably based on current physical characteristics rather than their long-term potential (22). This initiates a vicious cycle for the non-selected players, as they do not receive the same level of support and competition as their selected counterparts, making it even more challenging for them to “catch up” and to (re)enter the TID system (35). As a result, misjudgments in selections can lead to potentials being overestimated and actual “talents” being overlooked (3, 22). Therefore, it can be recommended that coaches at all selection levels be provided with objective data on the biological age of players (e.g., through somatic estimation equations or skeletal age measures) to validly consider maturation in their selection decisions. Estimates of players’ maturation based solely on the coaches’ eye may not be sufficiently accurate (71). Furthermore, a number of proposed countermeasures to mitigate relative age and maturation selection biases are the subject of ongoing debate (72, 73), including the employment of more handball-specific selection tasks (24, 74), raising awareness of coaches/scouts (73), player labeling (75), (relative) age quotas (73), and bio and birthday banding (22, 76, 77). It is of paramount importance to identify the selection levels at which the largest proportions of biases emerge to deploy these countermeasures in a targeted manner and enable them to unfold their full potential. In this study, selections for the NomCamp only marginally amplified already existing maturation and relative age selection biases. Given that no practically significant relative age selection biases can be observed at the club level in German handball within the same age groups as examined in this study (78), it can be inferred that relative age selection biases emerge somewhere between the initial selection levels (i.e., county/district level) and the SelCamp. To the best of our knowledge, there is an absence of research addressing the issue of maturation selection bias below the SelCamp. Thus, further research at lower and middle selection levels is necessary to elucidate the accumulation and persistence of maturation and relative age selection biases within the TID system.

## Data Availability

The data analyzed in this study is subject to the following licenses/restrictions: the data that support the findings of this study are available upon reasonable request and only with the permission of the data proprietor Deutscher Handballbund e.V. Requests to access these datasets should be directed to Jochen Beppler, jochen.beppler@dhb.de.
